# Social Isolation in Adolescence Disrupts Cortical Development and Goal-Dependent Decision-Making in Adulthood, Despite Social Reintegration

**DOI:** 10.1523/ENEURO.0318-19.2019

**Published:** 2019-09-20

**Authors:** Elizabeth A. Hinton, Dan C. Li, Aylet G. Allen, Shannon L. Gourley

**Affiliations:** 1 Graduate Program in Neuroscience, Emory University, Atlanta, GA, 30329; 2 Center for Translational and Social Neuroscience, Emory University, Atlanta, GA, 30329; 3 Yerkes National Primate Research Center, Emory University, Atlanta, GA, 30329; 4 Department of Pediatrics, Emory University, Atlanta, GA, 30329; 5 Department of Psychiatry, Emory University, Atlanta, GA, 30329

**Keywords:** fluoxetine, HA-1077, juvenile, orbital, Rho-associated coiled-coil containing kinase, stress

## Abstract

The social environment influences neurodevelopment. Investigations using rodents to study this phenomenon commonly isolate subjects, then assess neurobehavioral consequences while animals are still isolated. This approach precludes one from dissociating the effects of on-going versus prior isolation, hindering our complete understanding of the consequences of social experience during particular developmental periods. Here, we socially isolated adolescent mice from postnatal day (P)31 to P60, then re-housed them into social groups. We tested their ability to select actions based on expected outcomes using multiple reinforcer devaluation and instrumental contingency degradation techniques. Social isolation in adolescence (but not adulthood) weakened instrumental response updating, causing mice to defer to habit-like behaviors. Habit biases were associated with glucocorticoid insufficiency in adolescence, oligodendrocyte marker loss throughout cortico-striatal regions, and dendritic spine and synaptic marker excess in the adult orbitofrontal cortex (OFC). Artificial, chemogenetic stimulation of the ventrolateral OFC in typical, healthy mice recapitulated response biases following isolation, causing habit-like behaviors. Meanwhile, correcting dendritic architecture by inhibiting the cytoskeletal regulatory protein ROCK remedied instrumental response updating defects in socially isolated mice. Our findings suggest that adolescence is a critical period during which social experience optimizes one’s ability to seek and attain goals later in life. Age-typical dendritic spine elimination appears to be an essential factor, and in its absence, organisms may defer to habit-based behaviors.

## Significance Statement

Humans and rodents who experience early-life traumas or adversities appear to be prone to habit-based behaviors, often occurring at the expense of goal-oriented actions. Despite consistencies across species, how adversity, particularly during specific developmental periods, causes long-term behavioral biases remains unclear. Compounding this issue, many rodent investigations using social isolation to model adversity test mice or rats while they are isolated, making it difficult to dissociate the consequences of current versus developmental hardship. We reveal that mice with a history of social isolation during adolescence are biased toward habit-like behaviors, despite social reintegration in adulthood. Biases are linked with abnormalities in glucocorticoid tone and prefrontal cortical dendritic spine elimination during adolescence and were corrected by manipulating actin cytoskeletal regulatory factors.

## Introduction

The early-life social environment influences neurodevelopment and behavior. For example, social adversity during adolescence is associated with lifetime risk of depression in women (Thapar et al., 2012). In rodents, considerable efforts have revealed that social isolation during the postweaning period simplifies oligodendrocyte morphology in the prefrontal cortex (PFC), induces anxiety-like behavior, and impairs working memory (Palanza, 2001; Makinodan et al., 2012), but investigations most commonly isolate subjects, then assess neurobehavioral consequences while animals are still isolated. This approach precludes one from dissociating the effects of on-going versus prior isolation. Critical periods during which social experience impacts long-term neurobehavioral functions are still being identified.

During typical adolescence, prospective goal-oriented decision-making improves as individuals become more sensitive to the real or perceived consequences of their behaviors (Blakemore and Robbins, 2012). In the absence of goal awareness or updating, organisms may instead defer to habits – familiar behaviors that are stimulus-elicited and insensitive to goals. Also, during adolescence, PFC neurons undergo dramatic structural reorganization and synaptic remodeling. Some dendritic spines and synapses are stabilized and refined, whereas others, up to 50% in certain regions, are pruned (Bourgeois et al., 1994; Huttenlocher and Dabholkar, 1997; Shapiro et al., 2017). Individuals who experience early-life adversities have an increased incidence of behaviors that can lead to addiction and obesity as adults, and Patterson et al. (2013) provided evidence that these behaviors may result from an overreliance on outcome-insensitive habits. Complementary investigations in rodents linked stress-related failures in goal-oriented action selection and resulting biases toward habit-based behaviors to changes in prefrontal cortical dendrite and dendritic spine structure (Dias-Ferreira et al., 2009; Barfield et al., 2017). Nevertheless, the long-term effects of social experience during adolescence have not been thoroughly investigated, and mechanistic factors in adversity-induced habit biases, occurring at the expense of goal-directed action, are only beginning to be understood.

Goal-directed action (1) requires associating behaviors with their likely consequences (i.e., learning action-outcome contingencies), and (2) it must be motivated by the current value of outcomes. Expectations and action selection strategies must also be updated when action-outcome contingencies or outcome values change. Using multiple tasks and several independent cohorts of mice, we discovered that adult mice with a history of social isolation during adolescence could demonstrate sensitivity to instrumental contingencies, but were less able to update action-outcome response strategies as behaviors became more familiar, even despite normalization of the social milieu in adulthood. Instead, they deferred to habit-like modes of response, reminiscent of orbitofrontal cortical (OFC) inactivation across rodent and primate species (Gremel and Costa, 2013; Gourley et al., 2013a; Jackson et al., 2016; Zimmermann et al., 2018; Parkes et al., 2018; Whyte et al., 2019), as well as hyperactivation (artificial stimulation), which can cause compulsive-like behavior (Ahmari et al., 2013; Gillan et al., 2016; Pascoli et al., 2018). We found that a history of social isolation impoverished oligodendrocyte marker expression in this region and induced dendritic spine excess on layer V excitatory neurons, suggestive of failures in age-appropriate dendritic spine pruning. Based on these patterns, we then identified a sensitive period during adolescence when manipulating key regulators of the actin cytoskeleton, the structural lattice that forms the shape and plasticity of dendritic spines, can correct structural and behavioral defects caused by social poverty.

## Materials and Methods

### Subjects

A total of 452 C57BL/6 mice bred in-house from The Jackson Laboratory stock were used. For dendritic spine imaging, C57BL/6-back-crossed mice expressed *thy1*-derived YFP (H line; Feng et al., 2000). Mice were provided food and water ad libitum except during instrumental conditioning when mice were food-restricted to ∼90–93% of their original weight to motivate food-reinforced responding. Mice were maintained on a 12/12 h light/dark cycle (7 A.M. on). Females were used unless otherwise noted, a decision discussed in the next section. Mice used for postmortem measures were not subject to behavioral testing. Procedures were in accordance with the Emory University Institutional Animal Care and Use Committee.

### Social isolation

Mice were weaned at postnatal day (P)21–P22 and housed in single-sex groups of six to eight. At P31, mice were either rehoused in groups of six to eight with novel conspecifics, or housed in isolation, living in individual “shoebox”-style cages. All cages were positioned on racks that provided each cage, individually, with ventilation, minimizing the ability of mice to smell each other. Isolated mice lived on one rack and group-housed mice lived on another, minimizing visual contact. At P60, mice were socially reintegrated, housed in new cages with novel conspecifics, with each cage containing three to four previously isolated mice and three to four previously socialized mice.

In the one experiment using male mice, we did not socially reintegrate mice because humane concerns regarding high levels of aggression in C57BL/6 males preclude housing of unfamiliar adult males together (Simon, 1979).

### Corticosterone (CORT) ELISA

Trunk blood was collected following decapitation at P31, P39, P49, P60, and P82 in chilled Eppendorf tubes between 5:15 and 8:30 P.M. Tubes were centrifuged for 30 min at 4°C, and blood serum was extracted. CORT levels were determined by ELISA in accordance with manufacturer’s instructions (Enzo). Because this experiment used a between-subjects design, data points are connected by curve fits, rather than connected lines.

### Adrenal and thymus gland extraction

Immediately following euthanasia at P82, adrenal and thymus glands were exposed via mid-line dissection and extracted from surrounding adipose tissue. Weights represent both glands as a percentage of body weight.

### Immunostaining for 2',3'-cyclic-nucleotide 3'-phosphodiesterase (CNPase)

Mice were euthanized by rapid decapitation at P39 or P82, and brains were stored for 48 h in chilled 4% paraformaldehyde, then transferred to 30% w/v sucrose, and sectioned into 40-μm sections on a microtome held at –15°C. All immunohistochemical work used free floating sections. Before each step, the sections were washed 3X in PBS. Sections were blocked with PBS, 4% normal goat serum (NGS), and 0.3% Triton X-100 (Sigma-Aldrich) for 1.5 h at room temperature. Sections were then incubated at 4°C overnight in 2% normal goat serum, 0.3% Triton X-100, and primary antibody against CNPase (Millipore; 1:200). The following day, sections were incubated for 1 h at room temperature in 1% NGS and 0.3% Triton X-100, with Alexa Fluor 633 (Life Technologies; 1:500) serving as the secondary antibody.

Sections were imaged in a single session using a Nikon 4550s SMZ18 microscope with settings held constant. Fluorescence density was determined using ImageJ. A sampling area was drawn using *The mouse brain in stereotaxic coordinates* (Franklin and Paxinos, 2007) as reference to confirm that samples were collected from equivalent rostral-caudal, medial-lateral, and dorso-ventral positioning throughout. The sampling shape and area were held constant for each section. Imaging and scoring were completed by a single rater blinded to group. One brain from an isolated mouse generated values >2 SDs outside of the mean in four of the 13 brain regions tested. We excluded this mouse.

### Immunostaining for PSD95

Mice were euthanized by rapid decapitation at P82, and brains were stored for 48 h in chilled 4% paraformaldehyde, then transferred to 30% w/v sucrose, and sectioned into 40-μm sections on a microtome held at –15°C. All immunohistochemical work used free floating sections. Before each step, the sections were washed 3× in PBS. Sections were blocked with PBS and 0.3% Triton X-100 (Sigma-Aldrich), 2% NGS, and 1% bovine serum albumin (BSA) for 1.5 h at room temperature. Sections were then incubated 48 h at 4°C in 1× PBS and 0.3% Triton X-100, 0.3% NGS, 1% BSA, and a primary antibody against PSD95 (Cell Signaling Technology; 1:500). Tissue was then incubated for 1 h at room temperature in PBS and 0.3% Triton X-100, 1% NGS, and a secondary antibody Alexa Fluor 594 (Jackson ImmunoResearch; 1:400).

The ventrolateral OFC was located using *The mouse brain in stereotaxic coordinates* (Franklin and Paxinos, 2007). Images were collected in a single session using a Leica SP8 confocal microscope with a 63× oil-immersion 1.4 NA objective at a resolution of 1024 × 1024 pixels with a pinhole of 0.40 airy units, scanning speed of 100, and line averaging of 2.

Puncta count was quantified by a blinded rater using ImageJ software. Brightness and contrast settings were first adjusted consistently for all grayscale 8-bit images while leaving pixel values unchanged. Next, all images were adjusted using a threshold for signal intensity to maximize puncta signal and minimize background noise. These images were then converted into binary versions. The total number of PSD95+ puncta with sizes ranging 0.01- to 4.00-μm^2^ pixel units were then quantified from these binary images.

### Immunoblotting

Mice were rapidly decapitated at P82; brains were frozen at –80°C and then sectioned into 1-mm sections. The ventrolateral OFC was dissected by a single experimenter using a 1-mm tissue core. Tissue was homogenized by sonication in lysis buffer [100 μl: 137 mM NaCl, 20 mM tris-Hcl (pH 8), 1% igepal, 10% glycerol, 1:100 Phosphatase Inhibitor Cocktails 2 and 3 (Sigma), 1:1000 Protease Inhibitory Cocktail (Sigma)]. Protein concentrations were determined by Bradford colorimetric assay (Pierce), and 15 μg/sample was separated by SDS-PAGE on a 12% gradient tris-glycine gel (Bio-Rad). Following transfer to PVDF membrane, membranes were blocked with 5% nonfat milk.

Primary antibodies were anti-PSD95 (Cell Signaling; 1:1000), with anti-HSP-70 (Santa Cruz; 1:5000) serving as a loading control. Immunoreactivity was assessed using a chemiluminescence substrate (Pierce) and measured using a ChemiDoc MP Imaging System (Bio-Rad).

### Dendritic spine imaging and quantification

YFP-expressing mice were euthanized by rapid decapitation at P39 or P82. Fresh brains were submerged in chilled 4% paraformaldehyde for 48 h, then transferred to 30% w/v sucrose, followed by sectioning into 40-μm sections on a microtome held at –15°C. Unobstructed dendritic arbors running parallel to the surface of the section were imaged on a spinning disk confocal (VisiTech International) on a Leica microscope.

Z-stacks were collected with a 100× 1.4 NA objective using a 0.1-μm step size, sampling above and below the dendrite. Dendrites were collected from secondary branches within 50–150 μm of the soma. They were 11–85 μm in length, but primarily 20–25 μm. After imaging, we confirmed at 10× that the image was collected from the ventrolateral OFC. Collapsed z-stacks were then analyzed by a single blinded rater using ImageJ. Each protrusion ≤4 μm was considered a spine, and bifurcated spines were considered singular units.

To generate density values, spine number for each dendritic segment was normalized to the length of the segment. Six to eight dendrites/mouse were imaged and scored, with each mouse contributing a single density (the mean of its dendrites) to initial comparisons between groups by ANOVA. Additional subsequent analyses are described in the Statistics section.

### Surgery and viral vectors

Some experiments used intracranial placement of designer receptors exclusively activated by designer drugs (DREADDs). Group-housed mice were P31 at the time of surgery. Mice were anesthetized with ketamine/dexdomitor (75 + 1 mg/kg, i.p., MedVet). AAV8-CaMKII-HA-rM_3_D(Gs)-IRES-mCitrine or AAV8-CaMKII-GFP (UNC Viral Vector Core) was infused bilaterally (0.5 μl/side) at AP +2.6, ML ±1.2, DV –2.8. Infusions were delivered 0.05 μl/min, with the needle left in place for five additional minutes. The scalp was sutured, and mice were revived with Antisedan (1 mg/kg, i.p., MedVet). Following behavioral testing, mice were euthanized, brains collected and stored in 4% paraformaldehyde for 48 h, then transferred to 30% w/v sucrose. Brains were sectioned at 40 μm, and fluorescence within the OFC was imaged and transposed onto images from the Mouse Brain Library (Rosen et al., 2000).

### Pharmacological treatments

Experiments using Gs-coupled DREADDs capitalized on previously validated procedures (Gourley et al., 2016). The DREADD ligand, clozapine-N-oxide (CNO; 1 mg/kg in a volume of 1 ml/100 g, i.p., Sigma-Aldrich), was dissolved in a 2% dimethyl sulfoxide (DMSO; Sigma-Aldrich) solution in 0.9% sterile saline and prepared on the day of injection. Injections were delivered immediately following the contingency degradation training session, described below, then mice were tested the next day, drug-free. Importantly, all mice, regardless of viral vector, received CNO, equally exposing all mice to any unintended consequences of the drug, such as conversion to clozapine (Gomez et al., 2017). Notably, we have also confirmed that the same dose of CNO does not itself have any effects in the same task, nor does it affect activity of the master cytoskeletal regulatory protein cofilin in the OFC (Whyte et al., 2019). Considering the field as a whole, the dose is low, minimizing the likelihood of off-target effects (Urban and Roth, 2015, their tables).

Other drugs were administered intraperitoneally daily in a volume of 1 ml/100 g from P39 to P47 with the exception of one treatment group, in which the administration period was P31–P39 as a comparison. These drugs were the following: 5 mg/kg fluoxetine (FLX) in PBS (LKT Laboratories; Doosti et al., 2013), 10 mg/kg fasudil in PBS (LC Laboratories; Swanson et al., 2017), 10 mg/kg RU38486 in 2% v/v EtOH and PBS suspension (Sigma-Aldrich; Swanson et al., 2013), and 40 mg/kg spironolactone (Sigma-Aldrich) in 2% v/v EtOH and PBS suspension.

### Behavioral assays

#### Instrumental response training

Mice were food-restricted to ∼90–93% of their original body weights and trained to nose poke for grain-based food reinforcers (20 mg, Bio-Serv) in Med-Associates operant conditioning chambers. Chambers were equipped with two nose poke recesses and a separate food magazine. Responding was reinforced using a fixed ratio 1 (FR1) schedule wherein 30 pellets were available for responding on the two distinct nose poke recesses, resulting in 60 pellets/session. In initial experiments (see schematics in figures), mice were reinforced with two separate pellets (e.g., left nose poke resulted in a purified grain pellet, while right nose poke resulted in a chocolate pellet). Next, both nose pokes were reinforced with the same pellet (purified grain), duplicating the results reported in the prior figure. Subsequent experiments used a single reinforcer, and response rates are represented as total nose pokes/minute. Mice acquired the responses within five to seven 70-min sessions (1/d).

#### Instrumental contingency degradation

A modified version of classical action-outcome contingency degradation was used (as per Swanson et al., 2013, 2017; Gourley et al., 2012a). In a 25-min “non-degraded” session, one nose poke aperture was occluded, and responding on the other aperture was reinforced using a variable ratio 2 schedule of reinforcement. In the 25-min “degraded” session, the opposite aperture was occluded, and reinforcers were delivered into the magazine at a rate matched to each animal’s reinforcement rate from the previous session. Responses produced no programmed consequences. In this case, only ∼7% of pellets are delivered (by chance) within 2 s following a response (Butkovich et al., 2015). Thus, the schedule of reinforcement associated with both responses changes, relative to training, but one response becomes significantly less predictive of reinforcement than the other. Sessions were counter-balanced between and within groups. The following day, both apertures were available during a 10-min probe test conducted in extinction. Preferential engagement of the response most likely to be reinforced is considered goal-directed, evidence of updating instrumental response strategies.

In accordance with the model of Dias-Ferreira et al. (2009) and others, we mapped the development of responding that is likely to be habitual, which is outcome insensitive. To accomplish this, goal we took advantage of evidence that random interval (RI) schedules of reinforcement bias responding toward habit-based behavior, particularly with prolonged experience. Following the “early test” after FR1 training, responding on both apertures was reinforced according to an RI-30-s schedule of reinforcement for four sessions, then the instrumental contingency degradation procedure was repeated (“test”). An RI-60-s schedule was then used for five sessions, and contingency degradation was repeated (“late test”). When RI training was used, “RI” is indicated in the response training curves, and breaks in the curves represent instrumental contingency degradation tests.

#### Extinction

Following the final probe test shown for each respective cohort of mice, the same mice were placed in the conditioning chambers (Med-Associates) the next day for 15 or 75 min, as indicated graphically, for 2 d. Responding in the absence of reinforcement was compared between groups.

#### Conditioned taste aversion and reinforcer devaluation

Mice were trained to nose poke as described. Then, mice were placed individually in clean cages with ad libitum access to the reinforcer pellets for 1 h. Mice were then injected with 0.15 M LiCl (40 ml/kg, i.p.; Quinn et al., 2007) to induce conditioned taste aversion. The process occurred six times (one pairing/day). When mice had been trained to acquire two unique pellets, we paired each mouse’s preferred pellet with LiCl, and the other pellet was paired six times with NaCl (40 ml/kg, i.p.) as a control condition. Pairings were alternated (1/d).

The following day, mice were returned to the conditioning chambers, and responding in extinction was monitored. When a single pellet was used, response rates were monitored for 10 min and compared to the final day of training. When two pellets were used, response rates were monitored for 15 min and compared between LiCl and NaCl conditions.

#### Cocaine-induced locomotor activity

Female mice were isolated during adolescence and treated with fasudil or vehicle from P39 to P47. At P60, isolated mice were housed with each other in groups of eight. (In other words, in this experiment assessing whether fasudil influences cocaine-induced locomotor sensitization, all mice were subject to isolation.) Mice were then injected daily with both cocaine (10 mg/kg, i.p.) and vehicle. Following each injection, mice were placed in locomotor monitoring chambers (Med-Associates) for 1 h. Injections were separated by ≥3 h. The order of injections was randomized between and within subjects. This protocol is sensitive to increases or decreases in cocaine-induced locomotor activity (Gourley et al., 2009).

#### Elevated plus maze

Naive socially housed female mice were injected with fasudil or vehicle 30 min before test. The maze consisted of two open arms (50 × 6.5 cm) and 2 “closed” arms with walls (50 × 6.5 × 15 cm) attached to a central platform (6.5 × 6.5 cm). The maze was elevated 65 cm from the floor. Mice were placed in the center of the maze under dim light, and exploratory behavior was recorded by a video camera suspended overhead for 6 min. Arm entries were calculated by a computer running Limelight software (Coulbourn).

#### Open field test

Naïve socially housed female mice were injected with fasudil or vehicle 30 min before test. Mice were placed in the center of a rectangular field (41 × 20 × 20 cm). Exploratory activity was videotaped under dim light for 6 min, and time spent in a 6 × 6 cm center square was recorded.

### Statistics

Blood serum CORT, response rates, exploration time on the elevated plus maze, and locomotor activity counts were compared by two-factor or three-factor ANOVA with repeated measures when appropriate. Tukey’s *post hoc* comparisons were applied to all possible comparisons following interactions or main effects between more than two groups, and significant *post hoc* comparisons are indicated graphically. Time spent immobile and in the center of the open field, as well as gland weights and PSD95 and CNPase quantification values, were compared by unpaired two-tailed *t* tests.

For dendritic spine analyses, dendritic spines were enumerated on six to eight dendrites per mouse. Densities were first compared by ANOVA, with each animal contributing a single value (the mean density of all of its dendrites). In a secondary analysis, we first evaluated all dendrites in the control group and divided them into thirds based on their densities. In this case, the highest third had densities ≥1.38 spines/μm; these dendrites were termed “spine-rich.” For each mouse, the proportion of spine-rich dendrites was then calculated. For instance, in a typical control mouse, a minority 33% of dendrites would be considered spine-rich. Group means were compared by one-sample *t* test against the expected one-third proportion, again with each mouse contributing a single value to avoid artificial power inflation. Significant differences indicate that a given group had greater or fewer spine-rich dendrites than would typically be expected. Notably, categorizing dendrites based on the control population, as opposed to predetermined cut-offs, accommodates naturally occurring variance between cohorts in tissue preparation and processing that can affect fluorescence.

Throughout all analyses, *p* ≤ 0.05 was considered significant, and values >2 SDs outside of the mean were considered outliers and excluded. Group sizes were based on power analyses of existing datasets. SPSS and SigmaStat were used.

## Results

Throughout the majority of these experiments, we isolated female mice from P31 to P60, most of the adolescent period in rodents (Spear, 2000; Green and McCormick, 2013). Control mice (i.e., socially housed mice) lived in groups of six to eight. At P60, we then re-housed all mice into new social groups to determine the manner in which a history of isolation, occurring only during adolescence, influences neurobehavioral outcomes in adulthood. Each cage contained three to four previously isolated and three to four previously socialized mice.

**Figure 1.**
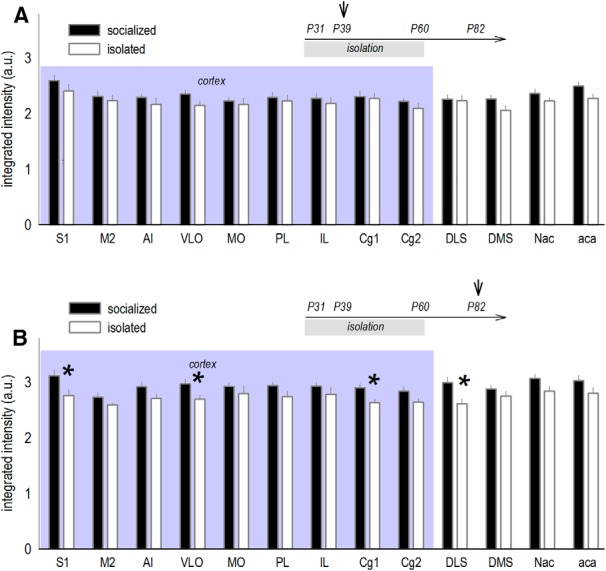
Effects of social isolation in adolescence on cortico-striatal CNPase during versus after isolation. Experimental timelines are inset, and shading highlights cortical regions. ***A***, Mice were socially housed or isolated starting at P31. CNPase was measured, given existing evidence that social isolation impoverishes oligodendrocyte morphology, and effects are persistent, despite social reintegration (Makinodan et al., 2012). At P39, CNPase did not differ between groups (*n* = 8–9/group). ***B***, Isolated mice were reintegrated into social groups at P60. At P82, previously isolated mice had decreased CNPase in multiple cortico-striatal subregions (*n* = 7–12/group). Mean ± SEM, *p < 0.05 versus socially housed control, replicated in two independent cohorts. S1, somatosensory cortex; M2, secondary motor cortex; AI, agranular insula; VLO, ventrolateral OFC; MO, medial OFC; PL, prelimbic PFC; IL, infralimbic cortex; Cg1, primary cingulate cortex; Cg2, secondary cingulate cortex; DLS, dorsolateral striatum; DMS, dorsomedial striatum; Nac, nucleus accumbens; aca, anterior commissure.

Although little is known regarding the long-term consequences of social isolation (specifically, those that manifest despite normalization of the social environment), important leads were reported by Makinodan and colleagues, revealing that social isolation durably simplifies oligodendrocyte morphology in the PFC (Makinodan et al., 2012). Thus, we initially validated our procedure by quantifying CNPase, an oligodendrocyte marker, throughout multiple cortico-striatal brain regions. During the social isolation period (at P39), CNPase levels did not differ between groups (all *p*s ≥ 0.067; Fig. 1*A*). At P82, however, more than three weeks following social reintegration, CNPase was diminished in several structures. These were: the ventrolateral OFC (*t*_(17)_ = –2.25, *p* = 0.038); prefrontal cortical Cg1 (*t*_(17)_ = –2.67, *p* = 0.016), the somatosensory cortex (*t*_(17)_ = –2.51, *p* = 0.022), and dorsolateral striatum (*t*_(17)_ = –2.67, *p* = 0.016; Fig. 1*B*). Thus, social experience during late adolescence (after P39) appears to support cortico-striatal CNPase.

### Social experience in adolescence optimizes instrumental response updating in adulthood

**Figure 2.**
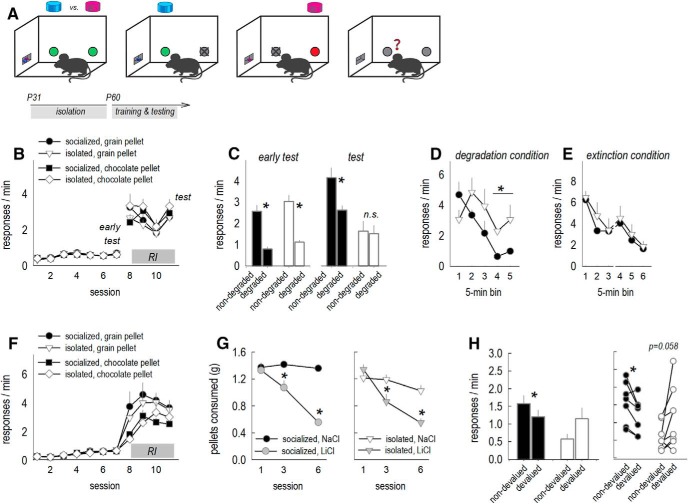
Social isolation in adolescence causes failures in instrumental response updating in adulthood. ***A***, A task schematic is provided: mice are trained to respond equivalently for two pellets. Then, the contingency between one nose poke and the associated pellet remains intact, while the other is degraded when that pellet is provided noncontingently. A brief probe test conducted in extinction gives mice the opportunity to demonstrate their response preference, if any, reflecting their ability to update response strategies. ***B***, Experimental timeline at top. Adult mice with a history of social housing or social isolation were trained to nose poke for two food reinforcers, with no group differences in response rates. Breaks in acquisition curves represent tests for sensitivity to instrumental contingency degradation. ***C***, In an early test, all mice decreased responding when the instrumental relationship associated with that response was violated (degraded). With additional training, however, previously isolated mice failed to update expectancies and deferred to habit-like behavior (test). ***D***, We also assessed response rates during the period of noncontingent pellet delivery. In this case, both groups decreased responding over time, but to a lesser degree in mice with a history of social isolation. ***E***, Meanwhile, responding in the absence of food pellets (extinction) was unaffected. The break in the *x*-axis represents the passage of 1 d (*n* = 8/group). ***F***, Separate mice were trained to nose poke for pellets. ***G***, LiCl was paired with each mouse’s preferred pellet, inducing conditioned taste aversion, while leaving consumption of the other pellet intact. ***H***, When returned to the conditioning chamber, control mice reduced responding for the now-devalued pellet. Meanwhile, mice with a history of social isolation continued to respond, failing to update response strategies. Right, Response rates of each mouse are plotted (*n* = 7–8/group). Bars and symbols represent mean ± SEM or individual mice, *p < 0.05.

CNPase patterns were notable because brain regions involved in both goal-directed response updating (e.g., ventrolateral OFC) and habitual behavior (e.g., dorsolateral striatum) were affected, even while others were spared (e.g., prelimbic and infralimbic cortices, respectively). These patterns raised the possibility that social experience during adolescence could have long-term effects on the ability of mice to appropriately balance goal-oriented actions versus habits. Action/habit decision-making can be defined using instrumental contingency degradation (Balleine and O’Doherty, 2010; Fig. 2*A*). Mice are first trained to generate two distinct nose poke actions for two distinct food reinforcers (e.g., left nose poke results in purified grain pellets, while right nose poke results in chocolate pellets). Then, food pellets associated with one nose poke are delivered non-contingently (instrumental contingency degradation), while the action-outcome contingency associated with the other behavior remains intact. Thus, one nose poke becomes significantly more predictive of reinforcement than the other. Next, mice have access to both nose poke recesses during a brief probe test conducted in extinction; the purpose of the probe test is to assess whether mice have updated action-outcome expectations, responding preferentially toward the intact instrumental contingency (considered a goal-directed action). Meanwhile, non-specific responding is considered habitual.

A history of social isolation did not impact response rates during training (no group × session interaction *F*_(10,140)_ = 1.28, *p* = 0.25, no effect of group *F* < 1), and pellet preferences were not detected (no group × pellet interaction *F*_(1,14)_ = 2.5, *p* = 0.14, no group × day × pellet interaction *F*_(10,140)_ = 1.86, *p* = 0.88, no effect of pellet *F* < 1), indicating that mice could learn to nose poke for pellets, and both pellets were preferred equivalently (Fig. 2*B*). In a probe test following instrumental contingency degradation, all mice initially favored the behavior most likely to be reinforced (early test, main effect of choice *F*_(1,14)_ = 100.5, *p* < 0.001, no effect of group or interactions *F*s ≤ 1; Fig. 2*C*), evidence of instrumental response updating.

We then tested sensitivity to instrumental contingencies following further training using RI schedules of reinforcement (Fig. 2*B*) that with time, can induce habitual behavior (Dickinson et al., 1983). A second contingency degradation test revealed that control mice retained sensitivity to instrumental associations, inhibiting a nose poke when reinforcement was unlikely. By contrast, previously isolated mice generated both responses equivalently during the probe test, despite instrumental contingency degradation (test, interaction *F*_(1,14)_ = 7.9, *p* = 0.01; Fig. 2*C*). When we compared responding during both probe tests together, we identified a group × response choice × probe test three-factor interaction (*F*_(1,14)_ = 5.5, *p* = 0.034), with *post hoc* comparisons indicating that response strategies differed between groups: While mice exposed to social isolation during adolescence were capable of learning about and updating instrumental associations, these mice more readily deferred to behaviors that are likely to be habitual than control mice.

We also assessed response rates during the period of noncontingent pellet delivery. Both groups decreased responding over time (main effect of time *F*_(4,56)_ = 6.8, *p* < 0.001; Fig. 2*D*), but to a lesser degree in the mice with a history of social isolation (interaction *F*_(4,56)_ = 3, *p* = 0.026; Fig. 2*D*). This pattern cannot obviously be attributable to differences in sensitivity to nonreinforcement, given that response extinction, tested following the final probe test, was unaffected (no group or interaction effects *F*s < 1; Fig. 2*E*).

The failure to select actions based on predicted outcomes is commonly associated with insensitivity to the *value* of an outcome (Balleine and O’Doherty, 2010). We thus next devalued one of two food reinforcers in separate mice subject to the same training procedures (FR1, followed by RI training). While response rates during training did not differ between groups (main effect, group × session, group × pellet, and group × pellet × session *F*s ≤ 1; Fig. 2*F*), rates associated with the chocolate pellet were modestly lower overall (*F*_(1,130)_ = 10.93, *p* = 0.006). Mindful of possible individual differences in pellet preferences, we next paired each mouse’s preferred pellet with LiCl, decreasing its value, while the other pellet was paired with NaCl, leaving its value intact. Pairings occurred in a separate environment relative to the training environment. LiCl pairings decreased intake over time as expected, while consumption of the NaCl-paired pellet did not change (session × pellet interaction *F*_(2,26)_ = 19.65, *p* < 0.001; Fig. 2*G*). A history of social isolation did not impact conditioned taste aversion (no group × session × pellet interaction *F*_(2,23)_ = 2.73, *p* = 0.11), although previously isolated mice consumed modestly less overall (main effect *F*_(1,13)_ = 6.32, *p* = 0.026).

When returned to the conditioning chambers, a pellet × group interaction was detected (*F*_(1,13)_ = 10.28, *p* = 0.007; Fig. 2*H*). Control mice generated higher response rates associated with the valued versus devalued reinforcer (*post hoc p* = 0.02). Meanwhile, response rates did not significantly differ between conditions in the previously isolated mice (*post hoc p* = 0.058). Inspection of individual mice (Fig. 2*H*, right) revealed that all but one control mouse inhibited responding associated with the devalued pellet, while several previously isolated mice generated higher response rates associated with the devalued pellet. This pattern is likely attributable to innate pellet preferences, given that we paired LiCl with each mouse’s preferred pellet. Thus, social isolation during adolescence weakens behavioral sensitivity to outcome value in adulthood.

**Figure 3.**
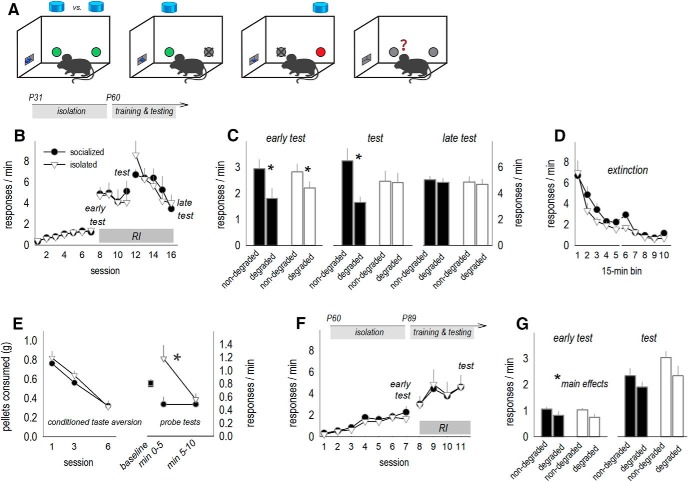
Social isolation in adolescence, but not adulthood, causes failures in instrumental response updating later in life. ***A***, A task schematic is provided: An instrumental contingency degradation task was used as in Figure 2, except both nose pokes resulted in the same type of pellet (represented here as a blue pellet). ***B***, Experimental timeline at top. Adult mice with a history of social housing or social isolation were trained to nose poke on two recesses for a single food reinforcer, with no group differences in response rates. Breaks in acquisition curves represent tests for sensitivity to instrumental contingency degradation. ***C***, In an early test, all mice decreased responding when the instrumental relationship associated with that response was violated (degraded). As in Figure 2, however, previously isolated mice failed to update response strategies with additional training, deferring to habit-like behavior (test). In a late test following extended training, all mice developed habit-like strategies (non-specific responding) as expected. ***D***, Response extinction was unaffected. The break in the *x*-axis represents the passage of 1 d (*n* = 12/group). ***E***, In a separate experiment, we decreased reinforcer value by pairing the food reinforcers with LiCl. LiCl induced conditioned taste aversion, as indicated by less food consumed over the course of multiple pairing sessions (left). When placed in the conditioning chambers, previously socialized mice inhibited responding relative to their pre-aversion baseline (before LiCl). By contrast, previously isolated mice initially responding robustly despite diminished outcome value before ultimately inhibiting their responding (right; *n* = 10–11/group). ***F***, Next, separate mice with a history of social isolation in adulthood were trained to nose poke for food reinforcers. ***G***, Unlike mice with a history of social isolation during adolescence, mice with a history of social isolation during adulthood retained outcome-sensitive response strategies (*n* = 6/group). “Main effects” refers to main effects of response type, regardless of group (no interactions). Mean ± SEM, *p < 0.05.

Mice can also be trained to respond on two separate nose pokes for a single food pellet (i.e., both left and right nose pokes are reinforced with a grain pellet; Fig. 3*A*). In this case, mice cannot use the distinct sensory properties of each unique reinforcer to help update response strategies. Using this procedure, we again found that a history of social isolation did not impact response rates during nose poke training (no group or interaction effects *F*s < 1; Fig. 3*B*). Following instrumental contingency degradation, all mice again initially favored the nose poke most likely to be reinforced (early test, main effect of choice *F*_(1,22)_ = 17.5, *p* < 0.001, no effects of group or interaction *F*s ≤ 1; Fig. 3*C*). A second test after RI training, however, revealed that previously isolated mice failed to update responding, generating both responses equivalently (group × response choice × probe test interaction *F*_(1,84)_ = 4.4, *p* = 0.04; Fig. 3*C*). This pattern duplicates our findings in the prior figure, and this single-reinforcer procedure was used for the rest of our instrumental contingency degradation experiments.

We took the opportunity to train these mice further using RI schedules to confirm that, with extensive experience, all mice would ultimately develop habit-like behavior in this procedure, as would be expected (late test, no group, response, and interaction effects *F*s < 1; Fig. 3*C*). Again, response extinction did not differ between groups, even when tested over a more prolonged period of time than in our initial experiments in Figure 2 (no group or interaction effects, *F*s < 1; Fig. 3*D*).

We next trained separate mice to respond for a single reinforcer, then devalued the food reinforcer by pairing it with LiCl. LiCl induced conditioned aversion as expected, with no differences between groups (no group or interaction effects *F*s < 1; Fig. 3*E*). When returned to the conditioning chambers, previously isolated mice nevertheless generated the food-associated response, apparently insensitive to reinforcer value, before ultimately inhibiting responding (group × time bin interaction *F*_(2,19)_ = 3.9, *p* = 0.05; Fig. 3*E*, right). Thus, even using a ratio schedule of reinforcement that would be expected to bias responding toward goal-sensitive response strategies, mice with a history of isolation display habit-like response tendencies before correcting them. Altogether, we thus used four assays (one- and two-reinforcer instrumental contingency degradation and one- and two-reinforcer devaluation) to reveal that social isolation during adolescence weakens the ability of adult mice update response strategies in an ever-changing environment, causing them to favor habit-like behavior.

To determine whether isolation-induced habit-like response biases are developmentally sensitive, we repeated the instrumental contingency degradation experiment, except we delayed isolation until P60. Following social reintegration, all mice acquired the reinforced responses without group differences (no group or interaction effects *F*s < 1; Fig. 3*F*) and were sensitive to changes in instrumental contingencies (early test, main effect of choice *F*_(1,10)_ = 9.6, *p* = 0.01, no effect of group or interactions *F*s ≤ 1; test, main effect of choice *F*_(1,10)_ = 5.5, *p* = 0.04, no effect of group or interactions *F*s ≤ 1; Fig. 3*G*). Thus, social isolation in adulthood did not obviously bias response strategies in the tests used here.

### Social experience during adolescence is necessary for age-typical glucocorticoid tone and dendritic spine densities

**Figure 4.**
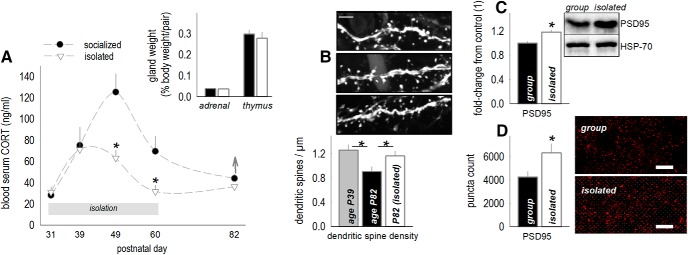
Social isolation induces glucocorticoid insufficiency in adolescence and dendritic spine and PSD95 excess in adulthood. ***A***, Blood serum CORT was measured during and after social isolation in adolescence. Social isolation blunted blood serum CORT, collected early in the active cycle when levels should be high. CORT normalized with social reintegration, such that adrenal and thymus gland weights were indistinguishable between groups by P82 (inset; *n* = 5–7/group). ***B***, Dendritic spine densities on excitatory neurons in deep-layer OFC were elevated following adolescent isolation. These densities, collected at P82, did not differ from those in typical socially housed adolescent (P39) mice, suggestive of a failure in dendritic spine pruning during adolescence (*n* = 6/group, with each mouse contributing a single value). Representative spines are above in the same order. Scale bar = 3 μm. ***C***, A history of social isolation also elevated OFC PSD95, as determined by Western blotting, and in an independent cohort of mice, (***D***) immunostaining (*n* = 6–8/group, with each mouse contributing a single value). Representative bands and images are adjacent. Scale bar = 10 μm. Mean ± SEM, *p < 0.05.

In adolescence, blood levels of the primary stress hormone CORT increase, then normalize as animals enter adulthood. We found that isolation induced corticosteroid insufficiency in late adolescence (starting at P39), such that blood CORT levels were low early in the active cycle (i.e., when they should be high). Unlike instrumental behavior, however, CORT normalized on social reintegration (day × group interaction *F*_(4,48)_ = 2.9, *p* = 0.03; Fig. 4*A*). Accordingly, adrenal and thymus gland weights were also typical at this time, corresponding to behavioral testing above (*t*_(11)_ = –0.5, *p* = 0.6; *t*_(11)_ = –0.59, *p* = 0.57; Fig. 4*A*, inset).

GR binding is necessary for dendritic spine turnover during adolescence (Liston and Gan, 2011), which culminates in dendritic spine pruning and lower densities in adulthood than adolescence. To determine whether social experience impacted age-typical dendritic spine elimination, we enumerated dendritic spines on layer V neurons in the ventrolateral OFC. Typical adult mice aged P82 had lower spine densities than typical adolescent mice aged P39, as expected, given that dendritic spines in this region are eliminated during adolescence (Gourley et al., 2012b; Milstein et al., 2013; Shapiro et al., 2017). Meanwhile, adult P82 mice with a history of social isolation had elevated densities, suggestive of failures in age-appropriate spine pruning (main effect *F*_(2,15)_ = 4.9, *p* = 0.02; Fig. 4*B*).

The protein PSD95 is a marker of stable, functional synapses (Berry and Nedivi, 2017). PSD95 was elevated in adult mice with a history of social isolation, as measured using Western blotting (*t*_(12)_ = 3.44, *p* = 0.005; Fig. 4*C*) and in an independent cohort, immunostaining (*t*_(14)_ = –2.25, *p* = 0.04; Fig. 4*D*). This pattern suggests that overabundant dendritic spines contained mature synapses.

### Artificially stimulating the ventrolateral OFC in healthy mice disrupts decision-making strategies

**Figure 5.**
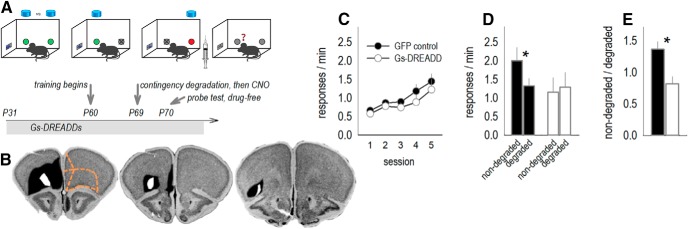
Artificial stimulation of the OFC causes failures in instrumental response updating. ***A***, The task used here was identical to that used in Figure 3. Experimental timeline: mice were infused with either a Gs-DREADD-expressing viral vector or a control viral vector into the OFC. Mice were trained to nose poke for food reinforcers. Next, one instrumental contingency was degraded. Immediately following, the presumed DREADDs ligand CNO was delivered to all mice. Response preferences were tested the next day, when mice were drug-free. ***B***, Histologic representation of viral vector spread is represented at right on images from the Mouse Brain Library (Rosen et al., 2000), with black representing the largest spread and white the smallest. Orange lines on the rostral-most section outline the prelimbic cortex (medial, top), the medial OFC (medial, bottom), and the ventrolateral and lateral OFC. Viral vector spread was largest in rostral sections, and more selective to the ventrolateral OFC in more caudal sections. ***C***, Mice were trained to nose poke for food reinforcers, with no differences between groups. ***D***, Gs-DREADDs mice failed to update expectations, deferring to habit-like behavior. ***E***, Response preferences were also calculated, with values >1 representing preference for the intact instrumental contingency. Again, Gs-DREADD mice failed to prefer the intact contingency. One value per group was considered an outlier and excluded (*n* = 12 control; *n* = 8 DREADDs). Bars and symbols represent mean ± SEM, *p < 0.05.

To explore the potential consequences of synaptic over-abundance in the OFC, we infused into typical, healthy mice CaMKII-driven viral vectors expressing Gs-coupled DREADDs and mCitrine (Fig. 5*A*,*B*). When activated, Gs-DREADDs artificially elevate neuronal excitability (Farrell and Roth, 2013). Infusions spanned the ventrolateral OFC in all mice, as intended (Fig. 5*B*). mCitrine was also detected in the medial OFC in a minority of mice, but this region is apparently not involved in sensitivity to instrumental contingency degradation (Bradfield et al., 2015).

Response training was not affected by DREADDs (no group effect *F*_(1,18)_ = 1.14, *p* = 0.3; no interaction effects *F*s < 1; Fig. 5*C*). CNO was then delivered to all mice, regardless of viral vector group, following instrumental contingency degradation, specifically following the session when one instrumental association was nullified. This timing was chosen based on evidence that inactivating the ventrolateral OFC at this same time occludes response updating in the same task (Zimmermann et al., 2018; Whyte et al., 2019). Also, experiments inactivating the ventrolateral OFC at multiple different time points in the testing procedure suggest that this region is essential for the stabilization, though not necessarily acquisition, of new instrumental memory (Zimmermann et al., 2018). Response preferences were tested when the mice were drug-free. We detected a group × response interaction (*F*_(1,18)_ = 7.29, *p* = 0.02), with *post hoc* comparisons indicating that control mice favored the response that remained likely to be reinforced, while Gs-DREADDs mice did not (Fig. 5*D*). To further understand response patterns, we also generated preference scores by simply calculating: non-degraded/degraded. In this case, values >1 indicate a response preference. Again, groups differed (*t*_(16)_ = –2.94, *p* = 0.01), with only control mice energizing the response that was likely to be reinforced (Fig. 5*E*). Thus, artificially activating the OFC is sufficient to impede response updating, causing mice to favor habit-like strategies.

### Inhibiting ROCK improves response updating and normalizes dendrite architecture

**Figure 6.**
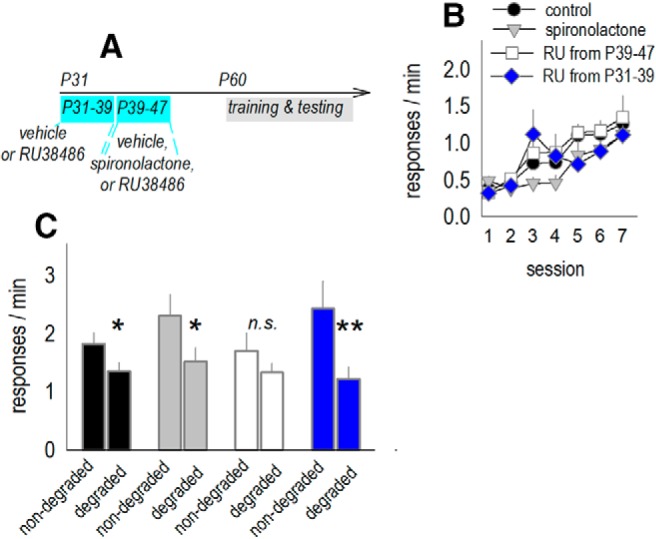
Identifying a sensitive period when GR binding in adolescence is necessary for response updating in adulthood. ***A***, Experimental timeline: to determine whether and when CORT insufficiency in adolescence was associated with response updating abnormalities in adulthood, typical, group-housed mice were treated with the GR antagonist RU38486 (RU) or the MR antagonist spironolactone during the same adolescent period when isolated mice experienced low CORT. A separate group of mice was treated with RU at an earlier period as a comparator. Mice were then tested for sensitivity to instrumental contingency degradation in adulthood. ***B***, Groups did not differ during response training. ***C***, However, mice exposed to the GR antagonist RU from P39 to P47 failed to update instrumental response strategies, indicated by insensitivity to instrumental contingency degradation (*n* = 15 total control; *n* = 7–9/drug group). Mean ± SEM, *p < 0.05, **p < 0.001. n.s., non-significant.

Our findings indicate that social isolation causes glucocorticoid insufficiency, dendritic spine excess, and biases toward habit-like behavior in adulthood. If these phenomena are connected, blocking GRs during adolescence should cause habit-like behaviors in adulthood. Also, normalizing dendritic spine densities in socially isolated mice should correct response biases. We next tested these predictions. First, we treated typical, socially housed adolescent mice with the GR antagonist RU38486 from P39 to P47, corresponding to a period when CORT levels diverged in typical versus isolated mice (Fig. 4*A*). Control mice received a vehicle solution, the MR antagonist spironolactone, or RU38486 during an earlier adolescent period, starting at P31, when isolation did not appear to impact CORT levels (Fig. 4*A*; experiment timeline in Fig. 6*A*).

As drug-free adults, mice were trained to nose poke for food reinforcers, with no significant differences between groups (no interaction *F*_(18,222)_ = 1.5, *p* = 0.08, no effect of group *F* < 1; Fig. 6*B*). In a test for sensitivity to instrumental contingency degradation, an interaction between group and response was detected (*F*_(3,36)_ = 3.5, *p* = 0.02; Fig. 6*C*). *Post hoc* comparisons revealed that mice exposed to the GR antagonist RU38486 starting at P39 failed to demonstrate response preferences, relying instead on habit-like behavior. Mice exposed earlier in development displayed intact response preferences, as expected based on prior reports (Swanson et al., 2013). Control mice and mice treated with the MR antagonist spironolactone also demonstrated response preferences (*post hoc p*s < 0.04). Thus, reducing GR binding during an early-life sensitive period impedes instrumental response updating later in life.

**Figure 7.**
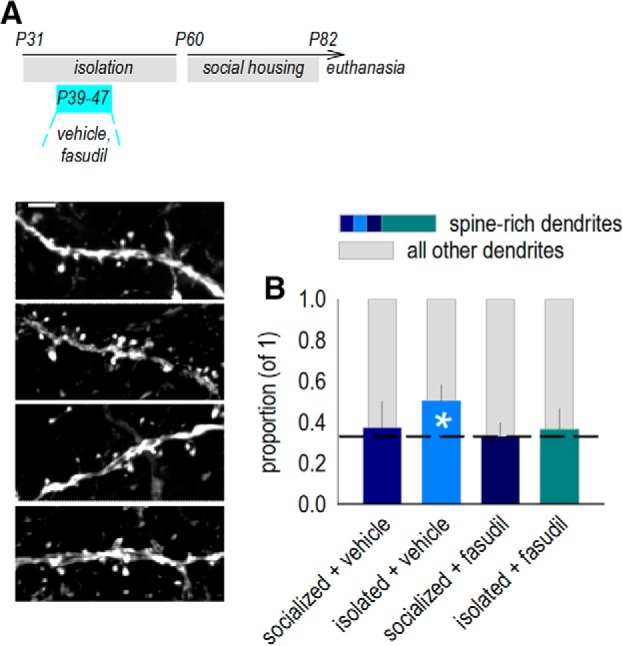
ROCK inhibition normalizes dendritic spine patterning in socially isolated mice. ***A***, Timeline: mice were socially housed or isolated in adolescence (P31–P60) and treated (from P39 to P47) with vehicle or fasudil. Mice were euthanized at P82, corresponding with the timing of behavioral experiments. ***B***, In typical mice, the proportion of spine-rich dendrites (colored in darker, blue tones) is smaller than the rest of the dendrite population, constituting 1/3 of all dendrites (dashed line). In mice with a history of social isolation, the proportion of spine-rich dendrites increased, such that they made up roughly half of all dendrites. Fasudil reinstated typical proportions, such that spine-rich dendrites again made up a minority of dendrites. Fasudil in typical, healthy mice did not appear to affect these proportions. Representative dendrites are adjacent in the same order (*n* = 6 mice/group, with each mouse contributing a single value). Scale bar = 3 μm. Mean ± SEM, *p < 0.05 versus one-third.

Next, we tested whether facilitating dendritic spine elimination in adolescence conferred behavioral effects. ROCK, particularly the ROCK2 isoform, is a neuronally enriched cytoskeletal regulatory factor; its inhibition can expedite dendritic spine elimination in multiple contexts, including adolescent prefrontal cortical development (Shapiro et al., 2019). Thus, we administered the ROCK2-favoring inhibitor fasudil during the same P39–P47 period, then euthanized mice as drug-free adults (Fig. 7*A*). Surprisingly, dendritic spine densities did not differ between any groups (*p*s ≥ 0.29; data not shown), potentially due to repeated injection stress during adolescence. Nevertheless, we identified an important pattern: We first divided dendrites from control mice into thirds based on their densities. This highest third had densities ≥1.38 spines/μm, which we term spine-rich. We then calculated the proportion of spine-rich dendrites for each mouse. In control mice, the mean proportion did not differ from one-third, as expected (*t*_(5)_ = 0.36, *p* = 0.73; Fig. 7*B*). In mice with a history of social isolation, spine-rich dendrites made up roughly half of all dendrites, significantly higher than is typical (*t*_(5)_ = 2.8, *p* = 0.04; Fig. 7*B*). Fasudil reinstated typical proportions, such that spine-rich dendrites again made up a minority of dendrites, not differing from the expected one-third (*t*_(5)_ = 0.37, *p* = 0.72; Fig. 7*B*). Fasudil did not obviously affect OFC dendrite architecture in socially housed control mice (*t*_(5)_ = 0.06, *p* = 0.96; Fig. 7*B*). Thus, fasudil normalized dendrite architecture in mice with a history of social isolation, ensuring that a minority of dendrites carried a large density of spines.

**Figure 8.**
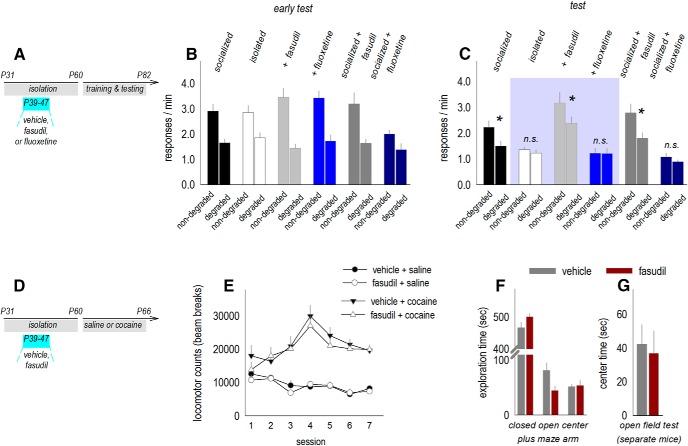
ROCK inhibition normalizes instrumental response updating following social isolation. ***A***, Timeline: mice were socially housed or isolated in adolescence (P31–P60) and treated (from P39 to P47) with vehicle, fasudil, or FLX. ***B***, As adults, mice were trained to nose poke for food reinforcers, and as in prior figures, all mice were initially sensitive to instrumental contingencies, inhibiting responding when it was unlikely to be reinforced (*n* = 19 total group control; *n* = 17 total isolation-housed control; other groups *n* = 8–10). ***C***, Subsequently, adult mice exposed to isolation during adolescence failed to update response strategies as expected (test). ROCK inhibition by fasudil rescued response strategies, while adolescent FLX promoted failures in response updating. Key comparisons are highlighted. ***D***, Timeline: mice were socially housed or isolated in adolescence (P31–P60) and treated (from P39 to P47) with vehicle or fasudil. As adults, locomotor sensitivity to repeated cocaine was tested. ***E***, Fasudil did not obviously impact the development of cocaine-induced locomotor sensitization (*n* = 8/group). Exploration of an elevated plus maze (***F***) and open field (***G***) were also not affected (*n* = 7/group, tested in separate cohorts of mice). Mean ± SEM, *p < 0.05. n.s., non-significant.

Next, we administered fasudil, again from P39 to P47, to separate mice for behavioral testing (Fig. 8*A*). Mice were either isolated or housed with conspecifics. As a comparator, other mice received the selective serotonin reuptake inhibitor (SSRI) FLX. We were motivated by evidence that artificial stimulation of the OFC can cause compulsive-like behavior, thought to have a habit component, that can be normalized by FLX in mature rodents (Ahmari et al., 2013).

As adults, mice were trained to respond for food reinforcers, and we assessed whether they could select actions based on their likely consequences. All groups initially engaged the action most likely to be reinforced following instrumental contingency degradation (early test, main effect of response *F*_(1,64)_ = 106, *p* < 0.001; no effects of isolation, treatment, or interactions; Fig. 8*B*). Further response training caused failures in response updating in previously isolated mice, as expected, which were blocked by fasudil (test, isolation × drug × response interaction *F*_(2,64)_ = 4.1, *p* = 0.02; *post hoc* comparisons in figure captions; Fig. 8*C*). Additional *post hoc* comparisons indicated that FLX had no effects in previously isolated mice and unexpectedly induced response updating failures in socially housed mice (Fig. 8*C*).

Interestingly, a history of fasudil appeared to increase food-reinforced response rates (Fig. 8*C*), which could conceivably be attributed to hypersensitivity to “reward.” However, the same dose of fasudil from P39 to P47 did not influence cocaine-induced locomotor sensitization, a classical measure of reward circuit plasticity (cocaine × fasudil and cocaine × fasudil × session *F*s < 1; Fig. 8*D*,*E*). This pattern was notable, given that higher doses can potentiate cocaine-induced locomotor sensitization (DePoy et al., 2013). We also assessed the behavioral effects of the same dose of fasudil while mice were adolescents, revealing no effects on exploration of an elevated plus maze (arm × group interaction *F*_(1,12)_ = 3.5, *p* = 0.09; Fig. 8*F*), nor open field (*p* > 0.05; Fig. 8*G*). This pattern was again notable, given that prolonged treatment in mature rodents has anxiogenic consequences (Greathouse et al., 2019). Thus, subchronic treatment of adolescent mice with a relatively low dose of fasudil does not induce locomotor abnormalities or anxiety-like behavior (see also Shapiro et al., 2019).

### Differential behavioral effects in male C57BL/6 mice

**Figure 9.**
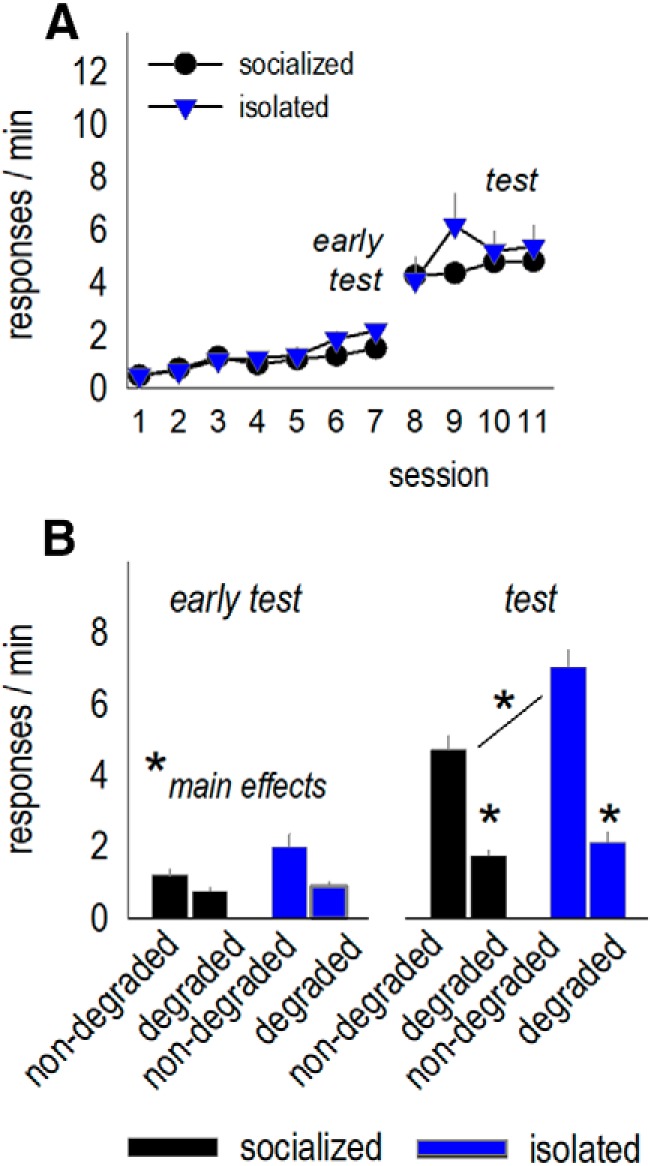
Intact instrumental response updating in male C57BL/6 mice subject to social isolation. ***A***, As in females, socially isolated males acquired the nose poke responses. ***B***, Unlike in females, males were able to select actions based on the likelihood of reinforcement, despite social isolation (*n* = 13 group; *n* = 11 isolated). Mean ± SEM, *p < 0.05.

All of our experiments above used female C57BL/6 mice. Males of this strain are aggressive and largely do not engage in “pro-social” behavior beyond the early adolescent period (Simon, 1979). For this reason, we anticipated different effects relative to females. Social isolation did not affect response rates during training (interaction *F*_(15,330)_ = 1.59, *p* = 0.075, no main effect of group *F* ≤ 1; Fig. 9*A*). During a first probe test following instrumental contingency degradation, a main effect of response choice indicated that all mice generated the response most likely to be reinforced (*F*_(1,22)_ = 20.66, *p* < 0.001, no interaction *F* < 1; Fig. 9*B*). In a second test, both groups again preferentially generated the response likely to be reinforced (unlike in females), and further, isolated males generating higher response rates (interaction *F*_(1,22)_ = 21.32, *p* < 0.001; main effect *F*_(1,22)_ = 209, *p* < 0.001; Fig. 9*B*). Thus, social isolation does not obviously cause response updating failures in male C57BL/6 mice.

## Discussion

The social environment during adolescence influences neurodevelopment. Investigations using rodents to study this phenomenon commonly isolate subjects, then assess neurobehavioral consequences while animals are still isolated. This approach precludes one from identifying critical periods when social experience molds the brain and behavior later in life. Makinodan et al. (2012) made important in-roads addressing this issue, reporting that social isolation during the postweaning period simplifies oligodendrocyte morphology in the PFC, despite social reintegration. Inspired by their report, we first measured CNPase, an oligodendrocyte marker, in mice exposed to social isolation during adolescence and socially reintegrated as young adults. CNPase was lower in multiple cortico-striatal regions involved in reward-related decision-making. CNPase patterns were notable because brain regions involved in goal-sensitive behavior (e.g., ventrolateral OFC) and habitual behavior (e.g., dorsolateral striatum) were affected, even while others involved in these processes were spared (e.g., prelimbic and infralimbic cortices, respectively). We then discovered that a history of social isolation weakened flexible instrumental response updating action in mice, causing them to favor habit-like behaviors, a bias linked to long-term effects on OFC neurobiology.

### Social interactions in adolescence optimize response flexibility in adulthood

Social isolation in adolescence induces anxiety- and depression-like behavior and reduces myelination throughout cortico-limbic structures (Green and McCormick, 2013). Decision-making capacity remains relatively unexplored, despite evidence that early-life adversity causes biases toward habit-based behaviors in humans (Patterson et al., 2013). Social isolation here decreased oligodendrocyte marker levels in multiple brain regions, as expected. One region was the OFC, a structure conceptualized as building a cognitive map of “task spaces,” allowing organisms to link behaviors and stimuli with anticipated outcomes (Wilson et al., 2014) and to update response strategies when expectations change (Sul et al., 2010).

To quantify decision-making behavior in mice with a history of social isolation, we used an instrumental contingency degradation procedure. Subjects are first trained to generate two reinforced behaviors. Then, the predictive relationship between one behavior and its outcome is violated by providing the associated food pellet noncontingently. Response inhibition is interpreted as evidence of response updating. We deployed two variants of the task, one using distinct reinforcers (two uniquely flavored pellets associated with two unique nose pokes), and another using a single reinforcer (one pellet is linked with both actions). The first iteration allows mice to integrate information regarding the sensory features of each outcome and connect them with the associated behavior. The second iteration is concentrated on instrumental contingency, but response patterns can generalize, which may or may not be habit-based. Both iterations are amenable to repeated testing, allowing us to define the nature of decision-making abnormalities, if any. Throughout, mice with a history of social isolation were initially capable of forming instrumental associations, inhibiting responding when one of the two trained behaviors was un-reinforced, presumably because some brain regions and neurobiological processes essential to goal-directed action were spared by social isolation. They, however, failed to update strategies as nose poking became more familiar. We interpret this pattern as reflecting a greater propensity to defer to habit-based response strategies, which are insensitive to action-outcome associations and resistant to updating. Responding was also insensitive to changes in outcome value, another marker of habits. Thus, social isolation in adolescence weakens goal-sensitive response updating behavior later in life, causing mice to favor habit-like response strategies, even despite social reintegration.

### Neurobiological factors

During adolescence, circulating CORT increases (Goldsmith et al., 1978; Meaney et al., 1985; Laviola et al., 2002; Silveri and Spear, 2004; Gunnar et al., 2009, Stroud et al., 2009; Fig. 4). Temporarily high CORT concentrations occupy low-affinity GRs and high-affinity MRs, coordinating high rates of cortical dendritic spine turnover and pruning during adolescence (Liston and Gan, 2011). We found that social isolation caused CORT insufficiency early in the wake cycle, when levels should be high (see also Sánchez et al., 1998), and concurrently, previously isolated adult mice retained adolescent-like spine densities. Further, PSD95, a marker of mature synapses, was elevated, suggesting that overabundant spines contained functional synapses (Berry and Nedivi, 2017).

To directly assess whether GR binding during adolescence affects decision-making in adulthood, we treated group-housed mice with a GR inhibitor at the same adolescent age when socially isolated mice experienced low CORT (approximately sixth week of life), causing habit-like response biases in adulthood. Thus, some minimal degree of GR activity in adolescence is necessary for optimal OFC function later in life, presumably acting by coordinating age-appropriate dendritic spine pruning (Liston and Gan, 2011). Naturally, other factors could be involved: Social isolation+reintegration triggers brain-derived neurotrophic factor *overexpression* in the PFC (Murinova et al., 2017), which has been associated with spinogenesis (Shapiro et al., 2017) and weakened goal-directed action (Gourley et al., 2012a). Also, upon social reintegration, socially housed mice appear to approach previously isolated mice less than the previously isolated mice approach them (Endo et al., 2018). These social imbalances normalize rapidly, however (1–2 d), so whether they would impact the long-term response biases identified here is unclear.

We next hypothesized that artificially “activating” the OFC of typical mice would induce habit-like response biases. Indeed, chemogenetic stimulation of CaMKII-expressing neurons in the ventrolateral OFC caused mice to fail to appropriately update response strategies; instead, they used habit-like behaviors. While this pattern was consistent with our expectations, it may seem unexpected to some readers, given that ventrolateral OFC inactivation also impedes response updating in this task in rats (Parkes et al., 2018), mice (Zimmermann et al., 2018), and nonhuman primates (Jackson et al., 2016). Importantly, repeated stimulation of the ventrolateral OFC causes compulsive-like behavior (Ahmari et al., 2013; Pascoli et al., 2018), thought to have a strong habit component (Gillan et al., 2016). We speculate that OFC stimulation intensifies connections that sustain compulsive behavior. Other possibilities are that stimulation is not causing compulsive-like behaviors, per se, but rather, interfering with essential task-related dendritic spine plasticity, including the pruning of certain spine types (Whyte et al., 2019). Also (or alternatively), OFC function may adhere to an inverted U-shaped curve, in which “too much” or “too little” activity impedes optimal response updating.

The actin cytoskeleton forms the structural lattice that supports the shape and plasticity of dendritic spines (Pontrello and Ethell, 2009). The protein ROCK influences cytoskeletal lability in large part by inhibiting cofilin (Maekawa et al., 1999; Koleske, 2013). ROCK inhibitors can augment neuronal structural plasticity, enhancing dendritic spine elimination associated with memory formation (Swanson et al., 2017), glutamate-mediated plasticity (Schubert et al., 2006), and adolescent development (Shapiro et al., 2019). Consistent with these patterns, the brain-penetrant ROCK inhibitor fasudil normalized dendritic spine excess in socially isolated mice here, decreasing the proportion of dendrites with high spine densities, and restoring typical proportions of spine-rich dendrites. Future investigations should identify the neurobiological properties of neurons vulnerable to dendritic spine over-abundance following social isolation (e.g., do they overexpress ROCK?). The ROCK inhibitor fasudil also corrected behavioral abnormalities, even compared to FLX, which is used to treat certain compulsive behaviors. Notably, FLX induced response updating failures. This outcome was surprising, given that serotonin has long been believed to sustain OFC function (Rogers et al., 1999a,b). Nevertheless, the enduring consequences of FLX in adolescence are not well understood.

### Concluding remarks

Female mice were used throughout the majority of experiments, given that in male mice, particularly aggressive C57BL/6 and CD-1 strains, single housing may not be a major adversity (Goldsmith et al., 1978; Bartolomucci et al., 2003; Arndt et al., 2009), and instead, advantageous, eliminating competition for reproductive opportunities. Isolation did not occlude response updating in C57BL/6 males, as expected. Meanwhile, exogenous CORT impairs response updating in males and females in the same task (Gourley et al., 2012a; Barfield et al., 2017), together suggesting that single housing uniquely affects male and female C57BL/6 mice.

We did not monitor estrus cycle to avoid stressor-related confounds from vaginal cell sampling, particularly given that CORT regulates dendritic spines on deep-layer cortical neurons (Liu and Aghajanian, 2008; Gourley et al., 2013b; Swanson et al., 2013; Barfield et al., 2017). Meanwhile, dendritic spines on deep-layer cortical neurons are considered less sensitive to sex than those in other structures such as the hippocampus (Muñoz-Cueto et al., 1991; Shors et al., 2001; see also Boivin et al., 2018). Additionally, our multiday behavioral experiments were replicated in multiple cohorts, and brain tissues were collected from two to three independent groups. Thus, it seems unlikely that rapid fluctuations in sex hormones due to the estrus cycle impacted our findings.

To conclude, social isolation during adolescence weakens age-typical dendritic spine elimination. Our findings are consistent with evidence that social play, other environmental stimuli, and stress hormones all contribute to neuron refinement during early-life critical periods (Zuo et al., 2005; Bell et al., 2010; Liston and Gan, 2011). Our finding that a ROCK inhibitory compound, delivered at a dose that normalizes dendritic spine excess, corrects deficiencies in flexible response updating provides empirical support for the supposition that neuronal refinement during adolescence is linked to complex, adaptive behavior in adulthood. A challenge in future investigations will be to comprehensively identify specific cell populations, projections, and cognitive domains durably affected by social adversity and develop clinically viable interventions to improve outcomes.

## Synthesis

Reviewing Editor: Karen Szumlinski, University of California at Santa Barbara Decisions are customarily a result of the Reviewing Editor and the peer reviewers coming together and discussing their recommendations until a consensus is reached. When revisions are invited, a fact-based synthesis statement explaining their decision and outlining what is needed to prepare a revision will be listed below. The following reviewer(s) agreed to reveal their identity: NONE. Note: If this manuscript was transferred from JNeurosci and a decision was made to accept the manuscript without peer review, a brief statement to this effect will instead be what is listed below.

I have reviewed this report, in conjunction with the prior round of reviews, and I find the article as it presently stands to be suitable for publication in eNeuro. The authors did a considerable amount of work to address the prior round of reviews and, I am of the impression that these edits have increased the strength of the report. I have no further comments at this time.
